# Renal Dysfunction was an Independent Predictor of In-Hospital Death and Ventricular Rupture in Patients With Acute Myocardial Infarction

**DOI:** 10.4021/cr184w

**Published:** 2012-05-20

**Authors:** Masayuki Goto, Eiji Oda, Hirooki Matsushita, Ken Takarada, Makoto Tomita, Atsushi Saito, Koichi Fuse, Satoru Fujita, Yoshio Ikeda, Hitoshi Kitazawa, Minoru Takahashi, Masahito Sato, Masaaki Okabe, Yoshifusa Aizawa

**Affiliations:** a Cardiovascular Center, Tachikawa Medical Center, Nagaoka, Japan; b Medical Check-up Center, Tachikawa Medical Center, Nagaoka, Japan; c Department of Research and Development, Tachikawa Medical Center, Nagaoka, Japan

**Keywords:** Acute myocardial infarction, In-hospital death, Ventricular rupture, Renal dysfunction

## Abstract

**Background:**

Apart from the severity of myocardial infarction and coronary artery disease, several predictors of in-hospital death (In-HD) are suggested in patients with acute myocardial infarction (AMI).

**Methods:**

We investigated predictors of In-HD and ventricular rupture (VR) including ventricular septal rupture (VSR) and free wall rupture (FWR) with stepwise multivariable logistic regressions in 1,042 patients admitted to our Cardiovascular Center within 48 hours from symptom onset for the first attack of AMI.

**Results:**

In-HD, VSR, and FWR were observed in 78 cases (7.5%), 14 cases of which 13 cases were In-HD, and 13 cases of which 6 cases were In-HD, respectively. Apart from the disease severity, age and renal dysfunction (RD) defined by estimated glomerular filtration rate of lower than 60 mL/min/ 1.73 m^2^ were independent positive predictors of In-HD (the odds ratios (ORs) (95% confidence interval (CI)): 1.04 (1.01 - 1.06) P = 0.0069 and 5.75 (3.12 - 10.59) P < 0.0001, respectively) and hypercholesterolemia was an independent negative predictor for In-HD (OR (95% CI): 0.34 (0.17 - 0.67) P = 0.0017). After including the categories of coronary disease, ventricular rupture, and ejection fraction in predictors, RD remained an independent predictor of In-HD (OR (95% CI): 6.65 (2.67 - 16.60) P < 0.0001). Age (OR (95% CI): 1.07 (1.02 - 1.12) P = 0.0064), RD (OR (95% CI): 2.77 (1.18 - 6.49) P = 0.019), and diabetes (OR (95% CI): 2.52 (1.12 - 5.71) P = 0.026) were independent predictors of VR.

**Conclusions:**

RD was an independent predictor of In-HD and VR in patients with initial AMI.

## Introduction

The MIYAGI-AMI registry, a data base from a population of 2 million in northeastern Japan, proved that in-hospital death (In-HD) of acute myocardial infarction (AMI) has markedly decreased from 20% in 1979 to 8% in 2008 [1]. Similarly in a US population-based study including 1,703 patients aged 25 - 54 years and hospitalized with initial AMI, In-HD and 30-day mortality decreased by approximately 50% (P = 0.04) during 15 annual periods from 1975 through 2005 [2]. Advanced therapy, especially percutaneous coronary intervention (PCI), appears to have contributed to the reduction in In-HD [3].

To further decrease early mortality of AMI, the prevention and treatment of ventricular rupture (VR) including ventricular septal rupture (VSR) and left ventricular (LV) free wall rupture (FWR) need to be addressed [4, 5]. Japanese Coronary Intervention Study Group observed risk factors of In-HD including attempted PCI to left main coronary artery (LMC) disease, LV dysfunction, LMC disease, older age, multi-vessel disease, cerebrovascular disease, and diabetes as independent predictors [6]. In addition, many predictors of In-HD were pointed in AMI patients: female sex [7-12], diabetes [13, 14], obesity [15], and renal dysfunction (RD) [16-19].

In the present study, we investigated independent predictors of In-HD and VR in 1,042 patients with initial AMI.

## Subjects and Methods

### Study subjects

This retrospective observational study was based on the data from 1,042 AMI patients who were admitted to our Cardiovascular Center in 2000 - 2009. The inclusion criteria of AMI cases were the admission within 48 hours from symptom onset.

### The diagnosis of AMI

The diagnosis of AMI was based on the following findings: 1) The ST-segments of the ECG waveform were measured in the lead with the maximal amplitude 60 ms after the J-point. The segment was defined as elevated if it was ≥ 0.2 mV or ≥ 0.15 mV above the isoelectric line in men or women, respectively, for leads V2-V3 and > 0.1 mV for all other leads where the patient did not have a left bundle branch block or left ventricular hypertrophy; 2) Creatine kinase or troponin levels were elevated above the normal limit; 3) Coronary artery occlusion was confirmed by coronary angiography (CAG).

The exclusion criteria were a history of prior myocardial infarction, non-Japanese ethnicity, and the lack of body weight and height information.

Informed consent was obtained from each patient and the study was approved by the ethics committee in Tachikawa Medical Center.

### Data collection and definition of risk factors

Data were collected from clinical charts. BMI was calculated as body weight (kg) divided by square of height (m) and obesity was defined as BMI ≥ 25 kg/m^2^ [20]. Diabetes was diagnosed by pre-admission information or fasting glucose ≥ 7.0 mmol/L and/or hemoglobin A1c ≥ 6.5%. Hypertension was diagnosed by pre-admission information or systolic blood pressure ≥ 140 mmHg and/or diastolic blood pressure ≥ 90 mmHg in later stable hospital days after admission.

Hypercholesterolemia was diagnosed by pre-admission information or total cholesterol ≥ 5.7 mmol/L and/or LDL cholesterol ≥ 3.6 mmol/L. RD was diagnosed by estimated glomerular filtration rate (eGFR) < 60 mL/min/1.73m^2^ on admission. In eight patients whose serum creatinine levels were not measured on admission, RD was diagnosed by pre-admission information. Chest pain attacks only within one month before AMI attacks were excluded from angina pectoris.

### Statistical analysis

The mean age and BMI, and the prevalence of obesity, diabetes, hypertension, hypercholesterolemia, RD, current smoking, family history of AMI, angina pectoris, chronic heart failure (CHF), valvular heart disease, atrial fibrillation, hemorrhagic stroke, ischemic stroke, peripheral artery disease, aortic aneurysm, malignant disease, and other miscellaneous diseases were compared between the In-HD group and survivors.

Apart from the severity of coronary artery disease/myocardial infarction, stepwise multivariable logistic regressions were calculated using In-HD or VR as a dependent variable and age, female sex, RD, obesity, diabetes, hypertension, hypercholesterolemia, current smoking, family history of AMI, angina pectoris, CHF, valvular heart disease, atrial fibrillation, hemorrhagic stroke, ischemic stroke, peripheral artery disease, aortic aneurysm, malignant disease, and other miscellaneous diseases as initial independent variables. Similar logistic regressions using In-HD as a dependent variable were repeated in subjects younger than 75 years and in those younger than 69 years. The inclusion and exclusion criteria of stepwise logistic regressions were p values of 0.05 or lower and 0.1 or higher, respectively.

Multivariable odds ratios (ORs) of In-HD were also calculated for various degrees of RD (eGFR < 15, 15 - 29.9, < 30, and 30 - 59.9 mL/min/1.73m^2^) compared with eGFR ≥ 60 mL/min/1.73m^2^ excluding eight patients whose serum creatinine levels were not measured on admission.

Similar stepwise logistic regressions using In-HD as a dependent variable were calculated including the above mentioned variables plus VR (FWR and VSR) as independent variables in all patients, including above mentioned variables plus VR, the number of diseased coronary arteries, LMC disease, and PCI as independent variables in patients (n = 1,020) who underwent CAG, and including above mentioned variables plus VR, the number of diseased coronary arteries, LMC disease, PCI, and ejection fraction as independent variables in patients (n = 835) whose ejection fraction was measured.

Two means were compared by t-tests and three means were compared by Scheffe’s tests after ANOVA. Prevalence was compared by Chi-squared tests. P values of lower than 0.05 were considered statistically significant. All statistical analyses were performed with Dr SPSS-2 (IBM Japan, Tokyo, Japan).

## Results

### Patient characteristics

In 1,042 patients with initial AMI, 78 cases (7.5%) died in the hospital of which 19 cases (24.4%) died from ventricular rupture and 12 cases (15.4%) died from extra-cardiac causes; septic shock in three cases, pneumonia in two cases, mediastinitis, leg necrosis due to peripheral artery disease, cerebellar hemorrhage, acute renal failure with refusal of hemodialysis, hemorrhagic shock, iatrogenic leg necrosis, and nonoclusive mesenteric ischemia in one case each. FWR was found in 14 cases of which 13 cases died in the hospital and VSR was found in 13 cases of which 6 cases died in the hospital. CAG was performed in 1,020 cases (97.9%) and PCI was implemented in 873 cases (85.6% of CAG cases). Coronary artery bypass graft (CABG) was operated in 102 cases (10.0% of CAG cases). Heart surgeries other than CABG were performed in 30 cases, most of which were repairs for VR (FWR in 12 cases and VSR in 9 cases).

CAG findings and therapeutic interventions are shown in Table 1. One vessel disease was more frequently found in survivors than In-HD cases (61.6% vs. 28.2%, P < 0.0001) while three vessel disease and LMC disease were more frequently found in In-HD cases than survivors (15.4% vs. 8.6%, P = 0.046 and 17.9% vs. 5.2%, P < 0.0001, respectively). PCI was more frequently performed in survivors than In-HD cases (85.5% vs. 62.8%, P < 0.0001) and heart surgeries other than CABG were more frequently operated in In-HD cases than survivors (12.8% vs. 2.1%, P < 0.0001).

**Table 1:** Coronary Angiographical Findings and Therapeutic Interventions (%)

n	in-hospital death	survivors	P
	78	964	
coronary angiography	87.2	98.8	< 0.0001
no significant stenosis	1.3	1.7	0.801
one vessel disease	28.2	61.6	< 0.0001
two vessel disease	24.4	21.7	0.582
three vessel disease	15.4	8.6	0.046
left main coronary disease	17.9	5.2	< 0.0001
percutaneous coronary intervention	62.8	85.5	< 0.0001
intra-aortic balloon pumping	59.0	16.3	< 0.0001
percutaneous cardiopulmonary support	9.0	0.6	< 0.0001
extracorporeal ultrafiltration method	2.6	0.1	< 0.0001
temporary pacing	23.1	5.7	< 0.0001
implantable cardioverter-defibrillator	0.0	0.7	0.450
coronary artery bypass graft	11.5	9.6	0.589
other heart surgery^*^	12.8	2.1	< 0.0001

^*^ 70% of which were repairs for ventricular rupture.

### Predictors of In-hospital death

Means of age and BMI, and prevalence of other candidate predictors were presented in Table 2. The mean age was significantly higher in In-HD cases than survivors and the mean BMI and ejection fraction were significantly lower in In-HD cases than survivors. The prevalence of female sex, RD, valvular heart disease, CHF, ischemic stroke, other miscellaneous diseases, and ventricular rupture were significantly higher in In-HD cases than survivors and the prevalence of hypercholesterolemia was significantly lower in In-HD cases than survivors. The prevalence of diabetes and obesity were not significantly different between In-HD cases and survivors.

**Table2:** Candidate Predictors of in-Hospital Death

n	in-hospital death	survivors	P
	78	964	
age (years)	77.2 ± 10.1	68.1 ± 12.5	< 0.0001
BMI (kg/m^2^)	22.3 ± 3.2	23.4 ± 3.3	0.005
female sex	42.3	27.8	0.007
renal dysfunction	79.5	32.3	< 0.0001
obesity	20.5	29.5	0.093
diabetes	29.5	25.8	0.479
hypertension	57.7	63.1	0.345
hypercholesterolemia	14.1	40.1	< 0.0001
current smoking	32.1	43.0	0.059
family history of AMI	5.1	10.1	0.157
angina pectoris	17.9	15.6	0.577
valvular heart disease	7.7	3.1	0.033
chronic heart failure	7.7	2.8	0.018
atrial fibrillation	10.3	6.7	0.242
hemorrhagic stroke	2.6	2.0	0.720
ischemic stroke	21.8	12.3	0.017
peripheral artery disease	6.4	4.4	0.168
aortic aneurysm	0.0	2.4	0.401
malignant diseases	5.1	7.0	0.539
others miscellaneous diseases	44.9	31.0	0.012
ventricular rupture	24.4	0.8	< 0.0001
ejection fraction (%)^*^	33.5 ± 12.0	49.6 ± 10.6	< 0.0001

Mean ± SD or %, ^*^ in 835 patients whose ejection fraction was measured.

The final step multivariable ORs of In-HD excluding the disease severity from independent variables were presented in Table 3. The ORs (95% confidence interval (CI)) of In-HD for age (years), RD, and hypercholesterolemia were 1.04 (1.01 - 1.06, P = 0.0069), 5.75 (3.12 - 10.59, P < 0.0001), and 0.34 (0.17 - 0.67, P = 0.0017), respectively in all patients, those for RD, hypercholesterolemia, female sex, and hypertension were 12.63 (5.06 - 31.53, P < 0.0001), 0.25 (0.09 - 0.70, P = 0.008), 2.70 (1.09 - 6.66, P = 0.032), and 0.38 (0.16 - 0.87, P = 0.021), respectively in 647 patients younger than 75 years, and those for RD and hypertension were 13.62 (3.91 - 47.46, P < 0.0001) and 0.25 (0.08 - 0.84, P = 0.025), respectively in 452 patients younger than 69 years.

**Table 3:** Multivariable Odds Ratios^*^ of in-Hospital Death Apart From the Disease Severity

independent predictor	odds ratio	95% confidence interval	P
**in all subjects (n = 1,042)**			
age (years)	1.04	1.01 - 1.06	0.0069
renal dysfunction	5.75	3.12 - 10.59	< 0.0001
hypercholesterolemia	0.34	0.17 - 0.67	0.0017
**in subjects younger than 75 years (n = 647)**			
female sex	2.70	1.09 - 6.66	0.032
renal dysfunction	12.63	5.06 - 31.53	< 0.0001
hypercholesterolemia	0.25	0.09 - 0.70	0.008
hypertension	0.38	0.16 - 0.87	0.021
**in subjects younger than 69 years (n = 452)**			
renal dysfunction	13.62	3.91 - 47.46	< 0.0001
hypertension	0.25	0.08 - 0.84	0.025

^*^ The final stage odds ratios of stepwise multivariable logistic regressions using age, female sex, obesity, diabetes, hypertension, hypercholesterolemia, renal dysfunction, current smoking, family history of myocardial infarction, angina pectoris, chronic heart failure, valvular heart disease, atrial fibrillation, hemorrhagic stroke, ischemic stroke, peripheral artery disease, aortic aneurysm, malignant disease, and other miscellaneous diseases as initial independent variables.

Multivariable ORs (95% CI) of In-HD for various degrees of RD were presented in Table 4. The ORs (95% CI) of In-HD for eGFR < 15 (n = 23), 15 - 29.9 (n = 38), < 30 (n = 61), and 30 - 59.9 (n = 320) mL/min/1.73m^2^ were 4.00 (0.84 - 19.09, P = 0.082), 20.40 (7.81 - 53.31, P < 0.0001), 14.81 (6.19 - 35.40, P < 0.0001), and 4.98 (2.54 - 9.78, P < 0.0001), respectively compared with eGFR ≥ 60 mL/min/1.73m^2^. However, among 23 patients with eGFR < 15 mL/min/1.73m^2^, 14 patients (60.9%) were under hemodialysis.

**Table 4:** Multivariable Odds Ratios of in-Hospital Death for Various Degrees of Renal Dysfunction

renal dysfunction	odds ratio^*^	95% confidence interval	P
eGFR^#^ < 15 mL/min/1.73m^2^ (n = 23)	4.00	0.84 - 19.09	0.082
eGFR 15 - 29.9 mL/min/1.73m^2^ (n = 38)	20.40	7.81 - 53.31	< 0.0001
eGFR < 30 mL/min/1.73m^2^ (n = 61)	14.81	6.19 - 35.40	< 0.0001
eGFR 30 - 59.9 mL/min/1.73m^2^ (n = 320)	4.98	2.54 - 9.78	< 0.0001

^*^ compared with eGFR ≥ 60 mL/min/1.73m^2^, ^#^ estimated glomerular filtration rate.

The final step multivariable ORs of In-HD including the variables in Table 3 plus the disease severity as independent variables are shown in Table 5. The ORs (95% CI) for RD was 5.80 (3.01 - 11.16, P < 0.0001) including ventricular rupture as independent variables in all patients, 4.67 (2.36 - 9.24, P < 0.0001) further including coronary artery diseases and PCI as independent variables in 1,020 patients who underwent CAG, and 6.65 (2.67 - 16.60, P < 0.0001) further including ejection fraction as independent variables in 835 patients whose ejection fraction was measured.

**Table 5:** Multivariable Odds Ratios of in-Hospital Death Including the Disease Severity in Predictors

independent predictor	odds ratio	95% confidence interval	P
including ventricular rupture in independent variables in all subjects (n = 1,042)^*^			
age (years)	1.04	1.00 - 1.07	0.028
renal dysfunction	5.80	3.01 - 11.16	< 0.0001
ventricular rupture	35.64	13.04 - 97.45	< 0.0001
hypercholesterolemia	0.26	0.12 - 0.56	0.0006
current smoking	1.73	0.94 - 3.18	0.078
other miscellaneous diseases	1.62	0.94 - 2.77	0.082
**in subjects who underwent coronary angiography (n = 1,020)^#^**			
age (years)	1.04	1.01 - 1.08	0.015
renal dysfunction	4.67	2.36 - 9.24	< 0.0001
ventricular rupture	37.69	13.81 - 102.88	< 0.0001
hypercholesterolemia	0.24	0.11 - 0.55	0.0006
left main coronary artery disease	2.55	1.16 - 5.61	0.020
one vessel disease	0.35	0.18 - 0.66	0.001
current smoking	2.08	1.09 - 3.99	0.027
chronic heart failure	2.50	0.90 - 6.95	0.078
**in subjects whose ejection fraction was measured (n = 835)^$^**			
renal dysfunction	6.65	2.67 - 16.60	< 0.0001
ventricular rupture	6.93	1.22 - 39.48	0.029
ejection fraction (%)	0.88	0.85 - 0.92	< 0.0001
left main coronary artery disease	2.93	1.00 - 8.54	0.050
hypercholesterolemia	0.44	0.17 - 1.14	0.091

The initial independent variables: ^*^ those of Table 3 plus ventricular rupture, ^#^ those of ^*^ plus one vessel disease, two vessel disease, three vessel disease, left main coronary artery disease, and percutaneous coronary intervention, ^$^ those of ^#^ plus ejection fraction.

### Predictors of ventricular rupture

The multivariable stepwise regressions of VR were presented in Table 6. Age (OR (95% CI): 1.07 (1.02 - 1.12) P = 0.0064), RD (OR (95% CI): 2.77 (1.18 - 6.49) P = 0.019), and diabetes (OR (95% CI): 2.52 (1.12 - 5.71) P = 0.026) were independent predictors of VR.

**Table 6:** Multivariable Odds Ratios^*^ of Ventricular Rupture Apart From the Disease Severity

	odds ratio	95% confidence interval	P
age (years)	1.07	1.02 - 1.12	0.0064
female sex	2.26	0.97 - 5.30	0.060
renal dysfunction	2.77	1.18 - 6.49	0.019
diabetes	2.52	1.12 - 5.71	0.026

^*^ The final stage odds ratios of stepwise multivariable logistic regressions using age, female sex, obesity, diabetes, hypertension, hypercholesterolemia, renal dysfunction, current smoking, family history of myocardial infarction, angina pectoris, chronic heart failure, valvular heart disease, atrial fibrillation, hemorrhagic stroke, ischemic stroke, peripheral artery disease, aortic aneurysm, malignant disease, and other miscellaneous diseases as initial independent variables.

## Discussion

In the present study, we demonstrated that, apart from the severity of coronary artery disease/myocardial infarction, age and RD were independent positive predictors of In-HD and VR and hypercholesterolemia was an independent negative predictor of In-HD in patients with initial AMI. Female sex, other cardiovascular diseases, diabetes, and obesity were not independent predictors of In-HD. RD remained a strong independent predictor of In-HD even after adjusting for ventricular rupture, coronary artery diseases, PCI, and ejection fraction.

Shihara et al reported that the In-HD rate was 7.1% and that the most important risk factor for In-HD was attempted PCI to LMC disease and further independent risk factors for In-HD were LV dysfunction, LMC disease, older age, multi-vessel disease, cerebrovascular disease, and diabetes in PCI patients with AMI [6].

Vakili et al [7] and Andrikopoulos et al [8] reported that female sex was an independent predictor of In-HD in patients with AMI while Hirakawa et al [9] and Park et al [10] reported opposite results. Berger et al reported that female sex was an independent predictor of In-HD in AMI patients younger than 75 years but not in the older patients [11]. Simon et al also reported that female sex was an independent predictor of In-HD in AMI patients younger than 69 years but not in the older patients [12]. In our present study, female sex was an independent predictor of In-HD in AMI patients younger than 75 years but not in all patients or in those younger than 69 years. The number of younger or middle-aged female AMI patients may be too small to reveal a gender difference in the In-HD rate among those younger than 69 years.

Kvan et al [13] and Hirakawa et al [14] reported that, although diabetic patients had a higher In-HD rate than non-diabetic patients, diabetes per se was not an independent predictor of In-HD. Kosuge et al showed that multivariable ORs (95% CI) of In-HD for lean (BMI < 20 kg/m^2^), overweight (BMI 25 - 29.9 kg/m^2^), and obese (BMI ≥ 30 kg/m^2^) AMI patients were 1.92 (0.20 - 6.72, P = 0.11), 0.79 (0.12 - 7.56, P = 0.56), and 0.40 (0.43 - 2.55, P = 0.24), respectively compared to those with normal weight (BMI 20 - 24.9 kg/m^2^) [15]. These reports are in line with our present results that diabetes and obesity were not independent predictors of In-HD in patients with AMI.

We found no previous report which suggests hypercholesterolemia as an independent negative predictor of In-HD in patients with AMI. It is possible that this result might be due to pleiotropic effects of statin therapy [21-24]. Jone et al reported that myocardial infarct size was significantly reduced in statin-treated mice compared with vehicle-treated mice [21] and Bell et al reported that statin attenuates lethal reperfusion injury in mouse hearts [22]. Herrmann et al reported that pre-PCI statin therapy is associated with a reduction in the incidence of larger-sized, stenting-related myocardial infarctions [23]. Wright et al reported that administration of statin therapy during the first day of hospitalization for AMI was associated with lower In-HD rate [24]. These reports showing pleiotropic effects of statin therapy might explain the mechanisms of our present result that hypercholesterolemia was an independent negative predictor of In-HD in patients with initial AMI. In our present study, CRP, which is a marker of pleiotropic effects of statins, were significantly lower in those with hypercholesterolemia than those without (1.52 ± 3.72 vs. 2.23 ± 4.40 mg/dL, P = 0.024) among 746 patients whose serum CRP levels were measured on admission.

RD is reported to be an independent predictor in AMI patients undergoing PCI [16, 17, 25]. Kim et al reported that the multivariable OR (95% CI) of In-HD was 2.67 (1.44 - 4.93, P = 0.002) for patients with eGFR of 30 - 59 mL/min/1.73m^2^, and 4.09 (1.48 - 11.28, P = 0.006) for those with eGFR < 30 mL/min/1.73m^2^ compared to those with eGFR ≥ 60 mL/min/1.73 m^2^ in AMI patients [18]. Satoh et al reported that the multivariable ORs (95% CI) of In-HD were 8.26 (2.22 - 30.77) for patients with eGFR < 15 mL/min/1.73 m^2^ and 3.42 (1.01 - 11.61) for those with eGFR 15 - 29 mL/min/1.73 m^2^ compared with those with eGFR ≥ 60 mL/min/1.73 m^2^ in AMI patients [19]. Our present results are in line with these reports.

In the present study, age, RD, and diabetes were independent predictors of VR. Moreyra et al reported that, compared to patients with AMI without VSR, patients with VSR were older, more likely to be women, had increased rate of chronic renal disease, congestive heart failure, and cardiogenic shock, and were less likely to be hypertensive or diabetic (all p values < 0.0001) [4]. Sobkowicz et al reported that FWR was the cause of 20% of In-HD and patients with FWR were older than those without (72 vs 60 years, P < 0.0001), and women prevailed in those with FWR than without (62% vs 27% in the survivors, P < 0.01). We could not examine VSR and FWR separately because the numbers of the subjects were too small.

In conclusion, we demonstrated that RD was an independent predictor of In-HD and VR. Chronic kidney disease and cardiovascular disease are related to hypertension, dyslipidemia and endothelial dysfunction and the cardiorenal syndrome represents a complex molecular interplay of neurohumoral pathway activation, the renin angiotensin aldosterone axis, vascular inflammation, oxidative stress, cardiac hypertrophy and fibrosis in the heart and kidneys [26, 27]. The results of the present study which show the association between RD and In-HD and VR may reflect these complex aspects of cardiorenal syndrome.

### Limitations

The present study is a retrospective observational study, the information about pre-hospital medication was not available, and the number of patients with VR was small. Further studies are required to evaluate the present results.
